# Super Minimally Invasive Pulp Therapy for Severe Pulpitis: A Report of Two Cases

**DOI:** 10.7759/cureus.42505

**Published:** 2023-07-26

**Authors:** Yuki Kojima, Atsuki Yamaguchi, Hiroyuki Inoue

**Affiliations:** 1 Anesthesiology, Asahi General Hospital, Asahi, JPN; 2 Dental Anesthesiology, Kanagawa Dental University, Yokosuka, JPN; 3 Anesthesiology, Center Hospital of the National Center for Global Health and Medicine, National Research and Development Agency, Tokyo, JPN

**Keywords:** super minimally invasive pulp therapy, teeth vitality, pulpectomy, dental pulp, root fracture, periodontal disease, caries, tooth loss, preventive dentistry

## Abstract

In regions where preventive dentistry is widespread, tooth loss due to root fracture occurs approximately 10 times more frequently than that due to caries and periodontal disease. Root fracture is most likely to occur in non-vital teeth, where the dental pulp has been removed, often through a procedure known as pulpectomy. However, super minimally invasive pulp (SMIP) therapy has recently been reported as a novel treatment approach for pulpitis of any degree. In this study, SMIP therapy was performed to preserve the vitality of teeth in two patients with severe pulpitis.

Case one involved a 35-year-old man with a history of hypertension who presented with intense spontaneous pain in tooth #34. The pain was particularly severe while sleeping at night and on exposure to cold water or heat, but it was absent on percussion. Following the detection of cervical caries and severe pulp exposure, SMIP therapy was administered, and the tooth was subsequently restored using glass ionomer cement.

Case two involved an 18-year-old woman with no significant medical history who had deep caries in tooth #46. She experienced mild tooth pain when exposed to cold water, and examination revealed pulp exposure. We applied mineral trioxide aggregate over the dental pulp and restored the tooth using composite resin. The vitality of both teeth was maintained at the three-month follow-up.

To our knowledge, this is the first report of SMIP therapy for teeth with severe pulpitis. SMIP therapy is an innovative treatment that may cause a paradigm shift from conventional dental treatment.

## Introduction

A healthy life expectancy is influenced by oral health [1], and adequate oral health and functioning are crucial for maintaining a good quality of life [2]. The relationship between oral health and well-being is particularly evident in geriatric patients who have lost their teeth [3]. Therefore, taking daily precautions and attending regular checkups are essential for the early detection of oral diseases and the maintenance of oral health. Moreover, early detection enables less invasive treatment options for oral diseases [4,5].

Tooth loss can be caused by caries, periodontal disease, or root fracture [6]. In areas where preventive dentistry is common, tooth loss due to root fracture is approximately 10 times more frequent than that due to caries or periodontal disease [7]. Notably, root fracture is most likely to occur in non-vital teeth. Loss of tooth vitality is mostly due to pulpectomy, a procedure in which the dental pulp is removed. Non-vital teeth are five to seven times more likely to be lost than vital teeth [8,9]. Therefore, maintaining tooth vitality is essential for achieving good oral health [10]. To preserve the inflamed dental pulp, vital pulp therapies have been recommended, including direct pulp capping, indirect pulp capping, and pulpotomy. However, these therapies have limited indications and require a high diagnostic ability and significant experience. The one-year success rates of direct pulp capping, indirect pulp capping, and pulpotomy are 65-86%, 83%, and 76-98%, respectively [11-13]. The prognosis of each therapy varies, and establishing a definite prognosis is difficult. In cases of pulpitis, where patients often complain of severe pain, dentists tend to prioritize pulpectomy over vital pulp therapy to rapidly improve the chief complaint. Recently, super minimally invasive pulp (SMIP) therapy has been proposed as a novel treatment approach for pulpitis [14]. SMIP therapy is a groundbreaking method for treating pulpitis without surgically invading the dental pulp in any condition. In this report, we present the first two cases of severe pulpitis, wherein tooth vitality was successfully preserved using SMIP therapy, effectively avoiding the need for pulpectomy. Both patients provided informed consent, and the ethics review committee approved this study (approval number: 2023031419).

## Case presentation

SMIP therapy technique

**Figure 1:**
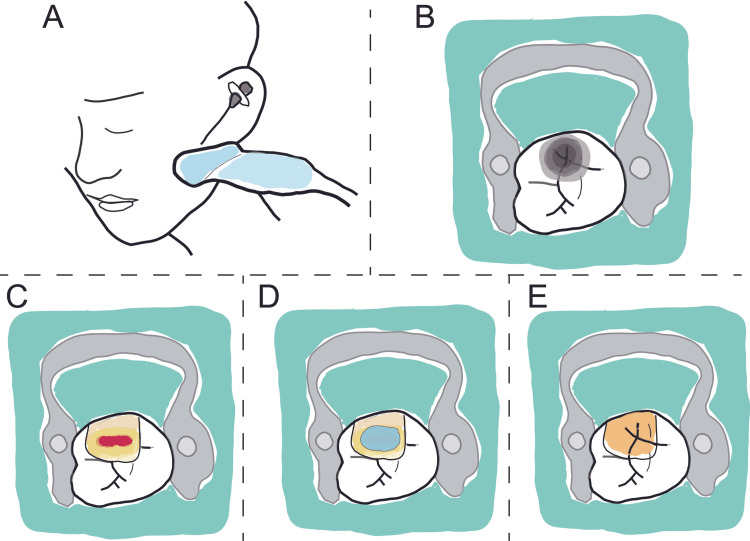
Schematic representation of (A) ultrasound-guided trigeminal nerve block. (B) Rubber dam isolation before removal of dental caries. (C) Dental pulp exposure after removal of dental caries. (D) Placement of mineral trioxide aggregate cement over the dental pulp. (E) Restoration of the tooth using composite resin.

First, the dental pulp was evaluated and confirmed to be vital. An intravenous line was secured after monitoring blood pressure and saturation and obtaining an electrocardiogram. The vital signs were normal during the administration of ampicillin hydrate (1 g). Midazolam was used to induce conscious sedation. Ultrasound-guided trigeminal nerve block (UGTNB) was performed using ropivacaine (Figure 1A). After injecting 6.6 mg dexamethasone, infiltration anesthesia was administered to the target teeth. Dental caries were removed under a rubber dam or cotton-roll isolation (Figure 1B). The tooth was washed with 300 mL (20 mL × 15 times) of strong acidic electrolyzed water (Oxilyzer; Miura Denshi, Nikaho, Japan) to clean the cutting surface. Disinfection and hemostasis of the exposed dental pulp were achieved using cotton balls soaked in hypochlorous acid (Figure 1C). Finally, the exposed dental pulp was sealed using mineral trioxide aggregate (MTA) cement (BioMTA; Morita, Tokyo, Japan) (Figure 1D). MTA cement is strongly alkaline and has potent bactericidal properties, which are essential for controlling pulpitis infection. The tooth was then restored using composite resin or glass ionomer cement (Figure 1E). The mechanisms of SMIP therapy are described as follows: UGTNB serves as an analgesic for severe pain, antibiotics address pulp infection, and steroids act as anti-inflammatory drugs. The goal of SMIP therapy is to simultaneously control pain, infection, and inflammation associated with pulpitis.

The dental pulp status was evaluated based on the presence of clinical symptoms using an electric pulp tester (YS-DT-A, RUENSHENG, China) and radiographs at the first visit and three months after SMIP therapy.

Case one

**Figure 2:**
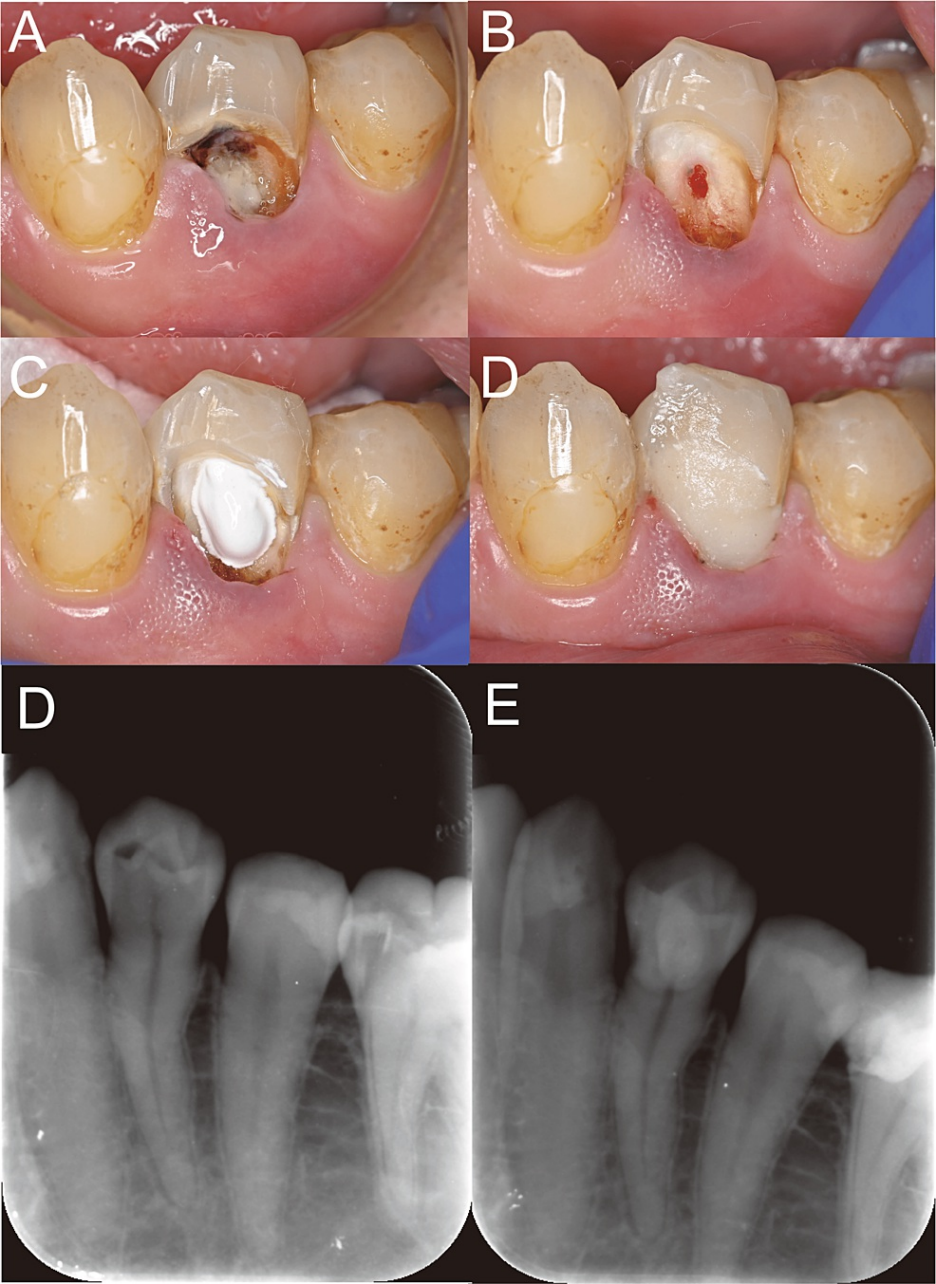
Clinical images and intraoral periapical radiographs of Case one. (A) Before treatment. (B) After removal of dental caries. (C) After placement of mineral trioxide aggregate cement over the dental pulp. (D) Preoperative radiograph. (E) Radiograph taken three months postoperatively.

A 35-year-old man (height: 170 cm; weight: 100 kg) with a history of hypertension experienced intense spontaneous pain in tooth #34 and consulted a nearby dentist one day before eventually coming to our hospital. After a consultation, he was advised to undergo a pulpectomy and subsequently referred to our hospital. He had severe pain during the night when he fell asleep, but no pain was present upon percussion. Tooth pain on exposure to cold water or heat was severe. Dental caries were observed in the cervical region (Figure 2A), and the dental pulp was exposed after the removal of a small amount of softened dentin (Figure 2B). After discussing with the patient his condition and treatment options, he strongly desired dental pulp preservation. Following an explanation of SMIP therapy, he selected this approach for severe pulpitis treatment. Ultrasound-guided inferior alveolar nerve block (UGIANB) was performed using 0.375% ropivacaine (6 mL). After the application of MTA over the dental pulp, the tooth was restored using glass ionomer cement (Figure 2C). No pain or discomfort was reported during surgery (Visual Analog Scale score, 0). Mild discomfort persisted until postoperative day three; however, the symptoms completely disappeared by postoperative day five. The results of the electric pulp test suggested that the vitality of the dental pulp was maintained at three months postoperatively. Additionally, radiographic examination revealed no abnormal findings (Figures 2D, 2E).

Case two

**Figure 3:**
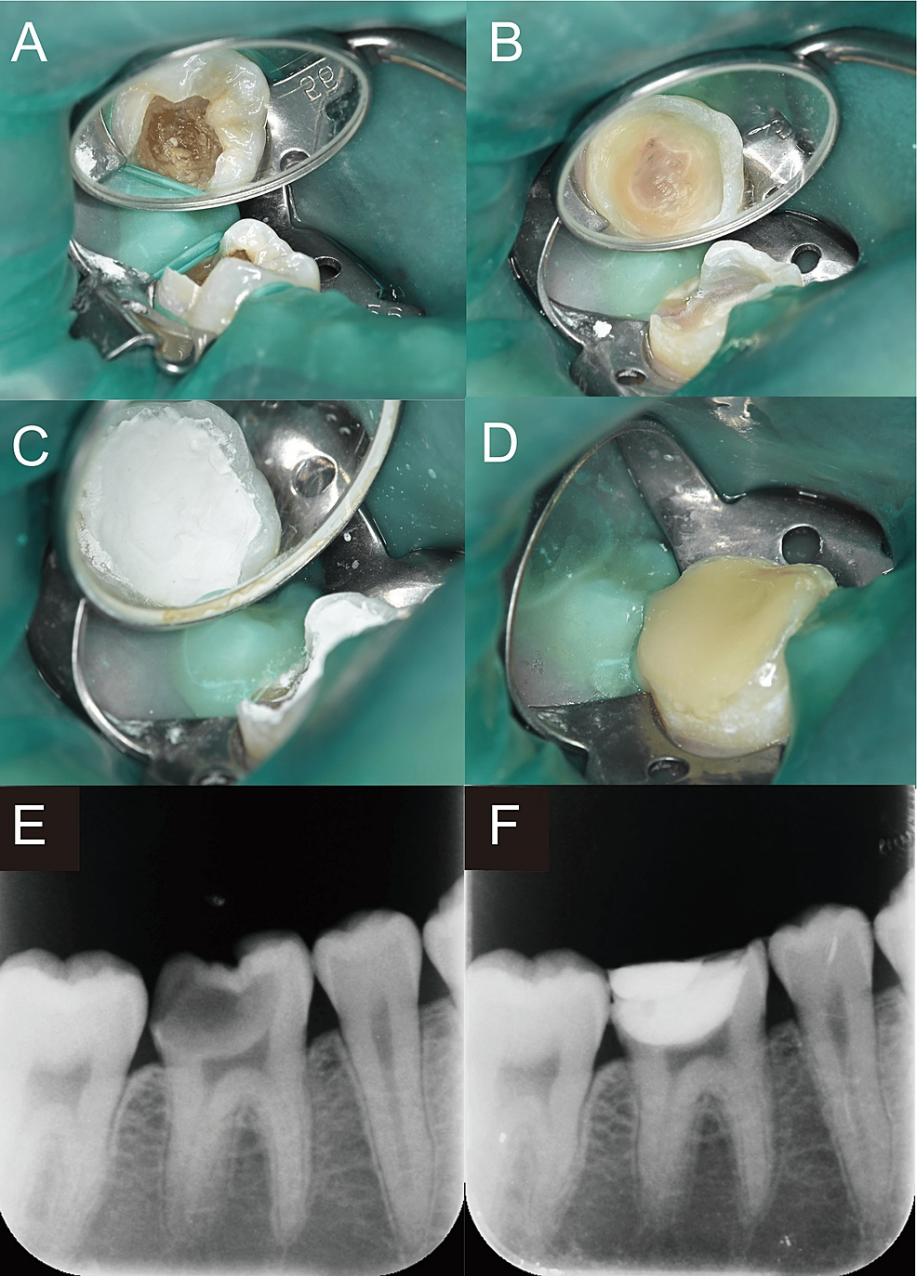
Clinical images and intraoral periapical radiographs of Case two. (A) Before treatment. (B) After removal of dental caries. (C) After placement of mineral trioxide aggregate cement over the dental pulp. (D) Preoperative radiograph. (E) Radiograph taken three months postoperatively.

An 18-year-old woman (height: 150 cm; weight: 50 kg) with no significant medical history was diagnosed with a deep carious lesion in tooth #46 by a nearby dentist, and the dentist recommended a pulpectomy (Figure 3A). However, the patient desired to preserve the dental pulp. She was subsequently referred to our hospital. Mild tooth pain occurred in contact with cold water. Under conscious sedation, UGIANB was performed for effective analgesia. All softened dentin was removed, and dental pulp exposure was observed (Figure 3B). After MTA application over the dental pulp, the tooth was restored using composite resin (Figure 3C). No pain or discomfort was reported during surgery (Visual Analog Scale score, 0). Postoperatively, the patient was asymptomatic, and radiographs showed no abnormalities (Figures 3D, 3E). At three months postoperatively, electric pulp testing revealed that the vitality of the dental pulp was maintained.

## Discussion

The dental pulp is a soft tissue containing blood vessels, connective tissues, and nerves. Caries surrounding the exposed dental pulp and preoperative spontaneous pain are signs of irreversible pulpitis [15]. Both patients in this study presented with spontaneous pain and caries surrounding the exposed dental pulp, confirming the presence of irreversible pulpitis. We believe that SMIP therapy will fundamentally change the concept of dental caries treatment as it does not require a specific diagnosis of the type of pulpitis and can be applied even in cases of severe pulpitis. With the concomitant administration of an effective analgesic, patients’ complaints can be significantly improved.

No diagnosis of pulpitis required

Pulpitis is classified as reversible or irreversible. Although there are several diagnostic methods, it is challenging to establish a reliable diagnosis in all cases [16]. Pain intensity, patient history, and responses to dental pulp sensitivity tests are the only clinical tools available to evaluate the severity of dental pulp inflammation [17]. However, in only a few cases, a definitive pathological diagnosis can be made based on clinical findings [18]. Moreover, the treatment protocol may vary depending on the extent of dental pulp inflammation and the infected area. Hence, inflammatory dental pulp diseases present a significant challenge for endodontists, requiring both practical experience and theoretical knowledge [17,19,20]. The dental pulp can be preserved in cases of reversible pulpitis, and irreversibly inflamed dental pulp tissue can heal if the pulpal infection and inflammation are controlled [21,22]. Recent position statements from the American Association of Endodontists and the European Society of Endodontology have emphasized that “pre-treatment diagnosis of irreversible pulpitis is not necessarily an indication for pulpectomy” [23,24], heralding a new era for minimally invasive treatment of teeth [25]. SMIP therapy can be applied if the dental pulp is vital, and it can be performed regardless of whether the dental pulp is reversibly or irreversibly inflamed, as well as the extent of infection. Therefore, considering the evidence accumulated to date, SMIP therapy is a rational treatment method.

Possibility of adaptation even in severe pulpitis

As pulpitis progresses, pulpal necrosis occurs. Partial dental pulp necrosis has been reported in many cases of pulpitis [21]. SMIP therapy can be applied to teeth with pulpitis accompanied by partial dental pulp necrosis. However, it is important not to introduce further infection via surgery in the area of dental pulp necrosis. We believe that removing the dental pulp or a large proportion of the hard tissue increases the risk of post-procedural infection and ultimately shortens the lifespan of the tooth.

Effective analgesia can improve patient complaints

Dentists are required to alleviate tooth pain due to pulpitis, which is the patient’s chief complaint. In the case of SMIP, the pain caused by pulpitis can be greatly reduced because pain relief is achieved with UGTNB [26]. The analgesic effect of UGTNB is strong and lasts for approximately 24-72 hours [27]. Steroids provide not only anti-inflammatory effects but also pain relief in pulpitis [28]. If pulpal infection and inflammation can be controlled under adequate analgesia, the patient can be treated painlessly. Sufficiently strong analgesia has been proven to be effective in surgery for malignant tumors and large-scale surgery for jaw deformity [29,30]. Our findings are consistent with those of a previous study, which demonstrated that favorable outcomes can be expected with sufficient analgesia even in dental caries treatment [26]. UGTNB can be expected to sufficiently improve the patient’s chief complaint of severe tooth pain due to pulpitis.

There are other reasons to avoid pulpectomy. Teeth undergoing pulpectomy will require root canal treatment, during which fine cracks may be generated depending on the equipment used [31]. These cracks may cause root fractures in the future [32]. In addition, the success rate of root canal retreatment is not high [33-36], and it is often necessary to repeat treatment. Repetition of root canal treatment increases the fragility of the tooth and leads to cracking fracture of the roots.

Dental infections are associated with a risk of serious acute illness [37]. Invasive osteomyelitis and neck abscesses may occur in patients with apical periodontitis [38]. Furthermore, dental infections, including apical periodontitis, can cause infective endocarditis. Thus, preservation of the dental pulp can maintain the immune function of the tooth and prevent future problems. Therefore, minimally invasive dental pulp protection treatment is necessary. SMIP therapy can be applied to several cases that would conventionally require pulpectomy. From a social point of view, preserving tooth vitality is meaningful because it reduces the number of treatments and associated medical expenses, eliminating the need for root canals and prosthetic treatments. However, it is important to note that these observations are based on case reports, and we cannot definitively conclude that SMIP therapy would be universally effective for all types of pulpitis. To clarify its definitive indications and effectiveness, further long-term studies involving a greater number of participants are needed.

Dental pulp regeneration therapy has been reported in recent years [39,40]. It is an innovative method and has been successful even in teeth that have undergone pulpectomy. However, this method can only be applied to a limited number of cases, and the prognosis remains unknown. Moreover, the long-term treatment lasting several years and the need for expensive medical equipment inevitably increase the burden on the patient [41,42]. Thus, it is in the best interest of the patient to maintain tooth vitality without damaging the dental pulp so that a pulpectomy is not required. The involvement of a dental anesthesiologist who can perform UGTNB and conscious sedation is essential for SMIP.

## Conclusions

SMIP therapy was performed in two vital teeth with severe pulpitis. Here, we report the details of the cases and actual techniques and outcomes of SMIP therapy. Although long-term prognosis data are unavailable, short-term follow-up data indicate satisfactory progress in both cases. SMIP therapy is an innovative treatment that may cause a paradigm shift in conventional dental caries treatment, and it may also be effective for irreversible pulpitis. It is important to prepare an environment for the generalization of SMIP therapy and the development of a more reliable method. Further large-scale clinical studies on the procedure, techniques, and outcomes of SMIP therapy are needed to verify its efficacy and long-term prognosis.
